# ΔPCO_2_ and ΔPCO_2_/C_(a−cv)_O_2_ Are Not Predictive of Organ Dysfunction After Cardiopulmonary Bypass

**DOI:** 10.3389/fcvm.2021.759826

**Published:** 2021-12-01

**Authors:** Sheng Zhang, Dan Zheng, Xiao-Qiong Chu, Yong-Po Jiang, Chun-Guo Wang, Qiao-Min Zhang, Lin-Zhu Qian, Wei-Ying Yang, Wen-Yuan Zhang, Tao-Hsin Tung, Rong-Hai Lin

**Affiliations:** ^1^Department of Critical Care Medicine, Taizhou Hospital of Zhejiang Province, Wenzhou Medical University, Linhai, China; ^2^Department of Cardiothoracic Surgery, Taizhou Hospital of Zhejiang Province, Wenzhou Medical University, Linhai, China; ^3^Evidence-Based Medicine Center, Taizhou Hospital of Zhejiang Province, Wenzhou Medical University, Linhai, China

**Keywords:** venous-to-arterial carbon dioxide difference, base excess, lactate, cardiopulmonary bypass, organ dysfunction

## Abstract

**Background:** Cardiac surgery is associated with a substantial risk of major adverse events. Although carbon dioxide (CO_2_)-derived variables such as venous-to-arterial CO_2_ difference (ΔPCO_2_), and PCO_2_ gap to arterial–venous O_2_ content difference ratio (ΔPCO_2_/C_(a−cv)_O_2_) have been successfully used to predict the prognosis of non-cardiac surgery, their prognostic value after cardiopulmonary bypass (CPB) remains controversial. This hospital-based study explored the relationship between ΔPCO_2_, ΔPCO_2_/C_(a−cv)_O_2_ and organ dysfunction after CPB.

**Methods:** We prospectively enrolled 114 intensive care unit patients after elective cardiac surgery with CPB. Patients were divided into the organ dysfunction group (OI) and non-organ dysfunction group (n-OI) depending on whether organ dysfunction occurred or not at 48 h after CPB. ΔPCO_2_ was defined as the difference between central venous and arterial CO_2_ partial pressure.

**Results:** The OI group has 37 (32.5%) patients, 27 of which (23.7%) had one organ dysfunction and 10 (8.8%) had two or more organ dysfunctions. No statistical significance was found (*P* = 0.84) for ΔPCO_2_ in the n-OI group at intensive care unit (ICU) admission (9.0, 7.0–11.0 mmHg), and at 4 (9.0, 7.0–11.0 mmHg), 8 (9.0, 7.0–11.0 mmHg), and 12 h post admission (9.0, 7.0–11.0 mmHg). In the OI group, ΔPCO_2_ also showed the same trend [ICU admission (9.0, 8.0–12.8 mmHg) and 4 (10.0, 7.0–11.0 mmHg), 8 (10.0, 8.5–12.5 mmHg), and 12 h post admission (9.0, 7.3–11.0 mmHg), *P* = 0.37]. No statistical difference was found for ΔPCO_2_/C_(a−cv)_O_2_ in the n-OI group (*P* = 0.46) and OI group (*P* = 0.39). No difference was detected in ΔPCO_2_, ΔPCO_2_/C_(a−cv)_O_2_ between groups during the first 12 h after admission (*P* > 0.05). Subgroup analysis of the patients with two or more failing organs compared to the n-OI group showed that the predictive performance of lactate and Base excess (BE) improved, but not of ΔPCO_2_ and ΔPCO_2_/C_(a−cv)_O_2_. Regression analysis showed that the BE at 8 h after admission (odds ratio = 1.37, 95%CI: 1.08–1.74, *P* = 0.009) was a risk factor for organ dysfunction 48 h after CBP.

**Conclusion :** ΔPCO_2_ and ΔPCO_2_/C_(a−cv)_O_2_ cannot be used as reliable indicators to predict the occurrence of organ dysfunction at 48 h after CBP due to the pathophysiological process that occurs after CBP.

## Introduction

Despite improvements in surgical technique, anesthesia, management, and postoperative care, cardiac surgery is still associated with a substantial risk of major adverse events (1). Early identification of risk factors and interventions reduce the occurrence of complications. Venous-to-arterial carbon dioxide (CO_2_) difference (ΔPCO_2_) is a parameter that reflects tissue hypoperfusion in critically ill patients who are insufficiently resuscitated (2). The PCO_2_ gap to arterial–venous O_2_ content difference ratio (ΔPCO_2_/C_(a−cv)_O_2_) has also been described as an indicator of the relationship between oxygen delivery (DO_2_) and oxygen consumption (VO_2_) (3). Although they have been successfully used to guide fluid resuscitation in patients with sepsis (4, 5) and predict prognosis of non-cardiac surgery patients (6, 7), the predictive value of CO_2_-derived variables after cardiopulmonary bypass surgery (CPB) is still controversial (8, 9). CO_2_-derived variables may be unable to inform on tissue ischemia because they do not track VO_2_ changes in cardiac surgical patients (10). Hyperlactatemia is related to tissue hypoxia but is also affected by factors such as catecholamines and metabolic rate. A previous study found that base excess (BE) is superior to lactate levels for the prediction of ICU mortality after cardiac surgery (11). Organ dysfunction is central to the pathogenesis of death and disability in critically ill patients, and the prognosis becomes worse as the number of failed organs increases. Therefore, it is more meaningful to use organ dysfunction as a clinical outcome variable because of the potential relationship. Although there are many studies on CO_2_-derived variables and clinical outcome, few studies have explored the prognostic value of CO_2_-derived variables basing on organ dysfunction. Organ dysfunction is considered to be an effective new outcome indicator in cardiac surgical patients, especially when the number of cases required is relatively small (12, 13).

The main objective of this study was to investigate relationship between CO_2_-derived variables and organ dysfunction occurring during the early phases after elective cardiac surgery with CPB. The secondary objective was to compare these variables with BE and lactate, which are usually associated with tissue hypoperfusion.

## Materials and Methods

We conducted a prospective observational study in a 39-bed mixed intensive care unit (ICU) of a university-affiliated Hospital. Our clinical trial was registered in the China Clinical Trial Registry (registration number: ChiCTR-ROC-17010727). Ethics approval was obtained from the Ethics Committee of Taizhou Hospital, Zhejiang Province. Written informed consent was obtained from all patients. The clinical trial started in February 2017 and ended in August 2018.

Adults (≥18 years old) admitted to ICU immediately after CBP, were included if an arterial line and a central venous catheter (confirmed by X-ray) had been inserted. After initiation of the trial, we removed one of the eligibility criteria: “Factors affecting the accuracy of cardiac output monitoring: aortic regurgitation, atrial fibrillation, aortic balloon counter pulsation, high dose norepinephrine,” because it was not necessary and would have reduced our sample size. The exclusion criteria were: emergency surgery, pregnancy, chronic renal insufficiency with dialysis, preoperative acute or chronic liver failure, hematologic diseases, misplacement of the central venous catheter, and death within 48 h.

Intraoperative and ICU management was conducted according to local protocols and international guidelines. Ventilator settings were as follows: control mode, tidal volume 6–8 mL/kg, positive end-expiratory pressure ≤ 5 cmH_2_O, respirations 14–16 times/min, fraction of inspired oxygen 40%. Analgesia was achieved with continuous infusion of fentanyl (0.5–10 mg/kg/h) targeting Critical Care Pain Observation Tool scores of 0–2. Anesthesia was maintained with a continuous infusion of propofol (1–4 mg/kg/h) targeting Richmond Agitation-Sedation Scale scores of−2–0. Vasoactive drugs and fluid infusions were adjusted according to mean arterial pressure of 60–70 mmHg. Infusion of red blood cells was prescribed as needed to maintain hemoglobin (Hb) concentration ≥9.0 g/dL. Crystalloid solution (Lactated Ringer's solution or 0.9% saline) or 20% albumin was added for volume expansion if necessary.

We prospectively collected preoperative, intraoperative, and postoperative variables, including clinical characteristics, duration of surgery, CBP duration, vital signs, fluid balance, vasoactive-inotropic score, routine blood tests, blood gas results, and clinical biochemistry. The specific data collection time points were at ICU admission and 4 h (H4), 8 h (H8), 12 h (H12), 24 h (H24), and 48 h (H48) after ICU admission. White blood cell count, hemoglobin concentration, platelet count, clinical biochemistry, chest X-rays, or CT scans were reviewed to evaluate organ function at ICU admission, H24, and H48. The length of stay in ICU, length of ventilator use, European System for Cardiac Operative Risk Evaluation II, Acute Physiology and Chronic Health Evaluation Score II (APACHE-II), and sequential organ failure score were recorded at ICU admission, H24, and H48. Paired arterial and central venous blood gases, arterial blood lactate, base excess (BE), and Hb levels were measured at ICU admission, H4, H8, and H12 using an automated analyzer (ABL800 Flex, Radiometer Medical Aps, Aakandevej 21, DK-2700 Bronshoj, Denmark).

Vasoactive-inotropic score (14) and CO_2_-derived and O_2_-derived variables were calculated as follows:

Vasoactive-inotropic score = dopamine (μg/kg/min) + dobutamine (μg/kg/min) + 10 × milrinone (μg/kg/min)

+ 100 × epinephrine (μg/kg/min) + 100 × norepinephrine (μg/kg/min) + 10,000 × vasopressin (μg/kg/min)


$$\begin{array}{c} \Delta {\text{PCO}}_{2}\;(\text{mmHg})={\text{P}}_{\left(\text{cv}-\text{a}\right)}\text{C}{\text{O}}_{2}={\text{P}}_{\text{cv}}\text{C}{\text{O}}_{2}-{\text{P}}_{\text{a}}\text{C}{\text{O}}_{2}\\ {\text{C}}_{\text{a}}{\text{O}}_{2}(\text{mL}/\text{L})=1.34\;\times \;{\text{S}}_{\text{a}}{\text{O}}_{2}\;\times \;\text{Hb}\\ {\text{C}}_{\text{cv}}{\text{O}}_{2}(\text{mL}/\text{L})=1.34\;\times \;{\text{S}}_{\text{cv}}{\text{O}}_{2}\;\times \;\text{Hb}\\ {\text{C}}_{\left(\text{a}-\text{cv}\right)}{\text{O}}_{2}\;(\text{mL}/\text{L})={\text{C}}_{\text{a}}{\text{O}}_{2}-{\text{C}}_{\text{cv}}{\text{O}}_{2}\\ \begin{array}{l} \Delta \text{PC}{\text{O}}_{2}/{\text{C}}_{\left(\text{a}-\text{cv}\right)}{\text{O}}_{2}(\text{mmHg}/\text{mL})={\text{P}}_{\left(\text{cv}-\text{a}\right)}\text{C}{\text{O}}_{2}/{\text{C}}_{\left(\text{a}-\text{cv}\right)}{\text{O}}_{2}=\\ \;({\text{P}}_{\text{cv}}\text{C}{\text{O}}_{2}-{\text{P}}_{\text{a}}\text{C}{\text{O}}_{2})/\;({\text{C}}_{\text{a}}{\text{O}}_{2}-{\text{C}}_{\text{cv}}{\text{O}}_{2}) \end{array}\\ \text{Oxygen}\;\text{extraction}\;\text{ratio}({\text{O}}_{2}\text{ER})(\text{\%})=\;1-{\text{S}}_{\text{cv}}{\text{O}}_{2}/{\text{S}}_{\text{a}}{\text{O}}_{2} \end{array}$$

The primary outcome variable was organ dysfunction at H48. Briefly, H48 organ dysfunction included: acute respiratory distress syndrome (PaO_2_/fraction of inspired oxygen <300 mmHg or PaO_2_ <60 mmHg requiring non-invasive ventilation or invasive mechanical ventilation support), acute kidney injury [Kidney Disease Improving Global Outcomes (KDIGO) level ≥1], acute tissue hypoperfusion (presence of tachycardia and hypotension associated with a central venous oxygen saturation <65%, cardiac index ≤ 2.2 L/min/m^2^), cardiac arrest (15) (cessation of cardiac mechanical activity, as confirmed by the absence of circulation signs), arrhythmia (15) (atrial fibrillation for ≥1 min was recorded, analyzed, and defined as “postoperative atrial fibrillation;” ventricular tachycardia and ventricular fibrillation were recorded by continuous ECG monitoring during the intensive care stay), acute neurologic dysfunction (stroke, seizure, persistent delirium, and Glasgow coma score below 12). According to the presence or absence of organ dysfunction at 48 h after CPB, the patients were divided into organ dysfunction group (OI) and non-organ dysfunction group (n-OI). The OI group was further divided into two subgroups: OI-1 including patients with one organ dysfunction, and OI-2 including patients with two or more organ dysfunctions.

### Sample Size

GPower software version 3.0.10 was applied to estimate required sample size for this study. This study used repeated-measure, between factors for the analysis. The study effect size was set at 0.25, power was set at 90%, alpha value was set at 0.05, and four -time measurements. Based on these, a minimum total sample of 108 subjects is required.

### Statistical Analysis

The statistical analysis was carried out using IBM SPSS 20 software. The Kolmogorov–Smirnoff test evaluated the normality of continuous variables and found that many continuous variables were non-normally distributed. Consequently, all continuous variables were expressed as median. The Mann–Whitney U test or Kruskal–Wallis test was used for group comparison, and repeated-measures ANOVA was used for intragroup comparison. Chi-square test (or Fisher's exact test, when appropriate) was used to compare categorical variables. GraphPad prism version 8.0.2 statistical software was used for boxplot charts.

According to the occurrence of organ dysfunction at H48, the patients were divided into n-OI and OI groups, with the OI group being further subdivided into OI-1 and OI-2 subgroups according to the number of organs with evidence of dysfunction at H48. Comparisons took place within the OI group, within the n-OI group, between the OI and n-OI groups, and between the n-OI and OI-2 subgroups. The ROC curve analysis was carried out to assess the predictive performance of the variables independently associated with H48 organ dysfunction. The area under the curve (AUC) was calculated based on their 95% confidence intervals (CIs).

In a subsequent analysis, variables were introduced into the logistic regression model if significantly associated with H48 organ dysfunction at the univariate analysis, when *P*-value was < 0.05. A Hosmer–Lemeshow test was used to assess the goodness of fit of the model.

## Results

### Clinical Characteristics and Prognosis

**Figure 1:**
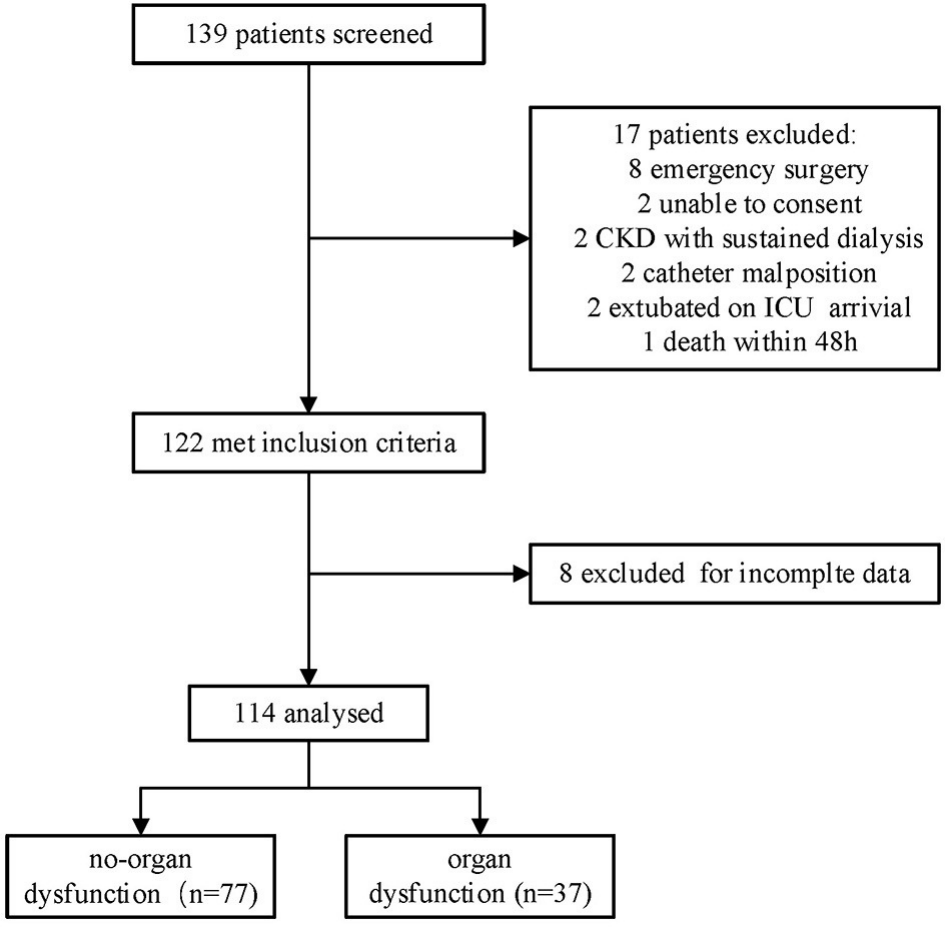
Study flow-chart. CKD, chronic kidney disease.

A total of 139 patients who fulfilled the criteria were screened and 114 patients were included (Figure 1). A total of 77 (67.5%) patients formed the n-OI group, and 37 (32.5%) patients comprised the OI group, of which 23.7% (27/114) had one organ dysfunction, 8.8% (10/114) had two or more organ dysfunctions. Compared to the OI group, the n-OI group had shorter length of stay in hospital [10.0 (8.5–14.0) vs. 13.0 (10.0–18.0) days, *P* = 0.01], shorter length of ICU stay [42.0 (24.0–48.0) vs. 47.0 (27.0–66.5) h, *P* = 0.02], and lower H24 and H48 sequential organ failure scores [3 (2–5) vs. 5 (4–7), *P* = 0.01] and [4 (3–5) vs. 5 (3–6), *P* = 0.02, respectively]. No statistical difference in the duration of mechanical ventilation was detected between the two groups of patients [19.0 (16.4–21.0) vs. 20.2 (18.5–22.8) h, *P* = 0.07] (Table 1).

**Table 1:** Clinical characteristics and prognosis of patients in the n-OI and OI groups (*N* = 114).

	**All (***N*** = 114)**	**n-OI (***n*** = 77)**	**OI (***n*** = 37)**	***P*** **-value**
Age (years)	58 (50–66)	56 (47–64)	63 (55–68)	0.01*
**Sex**				0.32
Male	52	38	14	
Female	62	39	23	
BMI (kg/m^2^)	22.7 (20.7–24.9)	22.2 (20.1–24.6)	23.4 (21.7–25.3)	0.048*
**Type of surgery**				0.58
Valve replacement (or plastic)	81	56	25	
CABG	3	1	2	
Combined surgery	13	8	5	
Other	17	12	5	
Duration of surgery (h)	4.3 (3.5–5.0)	4.0 (3.5–5.0)	4.5 (3.6–5.0)	0.31
CBP duration (min)	100 (78–135)	100 (80–135)	100 (75–145)	0.90
Aortic clamping duration (min)	65 (45–80)	65 (45–80)	60 (50–80)	0.94
Pacemaker (yes /no)	35/79	26/51	9/28	0.39
**Comorbidities**				0.38
Hypertension	45	27	18	
COPD	3	3	0	
Diabetes	8	3	5	
Stroke	6	4	2	
Chronic kidney disease	2	1	1	
Malignant cancer	2	0	2	
Gastrointestinal disease	4	3	1	
Coronary artery disease	1	0	1	
Vascular disease	3	2	1	
NYHA				0.93
I	8	5	3	
II	74	50	24	
III	30	21	9	
IV	2	1	1	
EuroSCORE	3 (2–5)	3 (1–4.5)	4 (1–5.5)	0.08
APACHEII (H24)	6 (4–8)	6 (4–7)	7 (4–10)	0.16
SOFA (H24)	4 (2–5)	3 (2–5)	5 (4–7)	0.01*
APACHEII (H48)	11 (9–15)	11 (9–14)	12 (9–16)	0.46
SOFA (H48)	3 (4-5)	4 (3–5)	5 (3–6)	0.02*
Length of ventilator (h)	19.5 (17.0–21.0)	19.0 (16.4–21.0)	20.2 (18.5–22.8)	0.07
LOS in ICU (h)	44.0 (24.9–48.5)	42.0 (24.0–48.0)	47.0 (27.0–66.5)	0.02*
LOS in hospital (days)	11.0 (9.0–15.0)	10.0 (8.5–14.0)	13.0 (10.0–18.0)	0.01*

*CABG, coronary artery bypass grafting; COPD, chronic obstructive pulmonary disease; NYHA, New York Heart Association functional classification; Euro SCORE, European system for cardiac operative risk evaluation; APACHE II, acute physiology and chronic health status score II; SOFA, sequential organ failure score; ICU admission, immediately after entering ICU; H24, 24 h after entering ICU; H48, 48 h after entering ICU. Continuous variables are represented by median (IQR). The Mann–Whitney U test was used for intergroup comparison. Categorical variables were used by Fisher's exact test*;

* *P < 0.05*.

### Dynamic Changes in CO_2_-Derived Variables in n-OI and OI Groups

The length of ventilator usage in all patients was 19.5 (range: 17.0–21.0) h. In order to reduce the influence of spontaneous breathing on CO_2_-derived variables after weaning, ICU admission, H4, H8, and H12 time points were selected for analysis.

**Figure 2:**
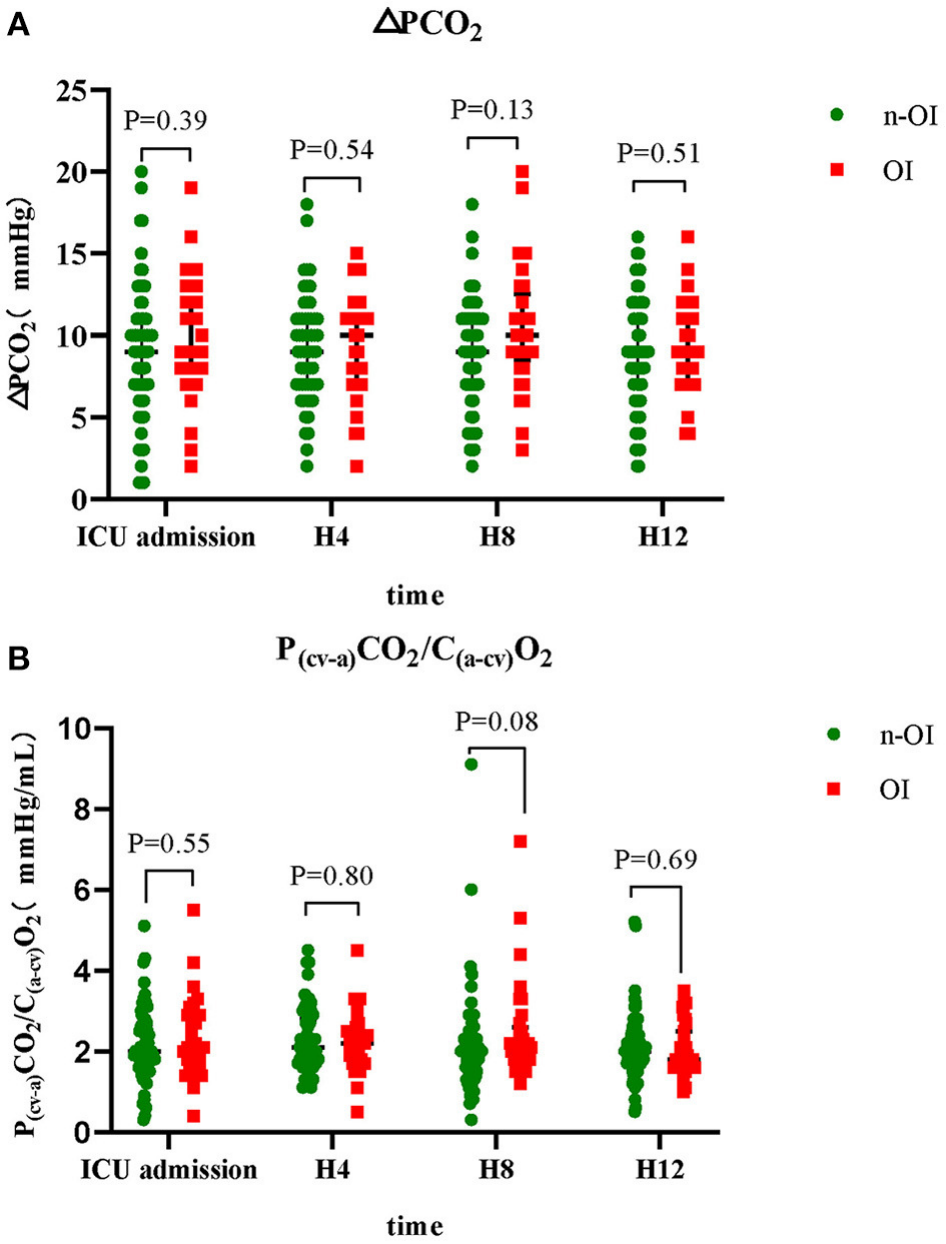
Dynamic changes of carbon dioxide-derived variables of n-OI and OI groups at the four time points. **(A)** central venous-to-arterial carbon dioxide difference (ΔPCO_2_) **(B)** ΔPCO_2_ to arterial-central venous oxygen content difference rate (ΔPCO_2_/C_(a−cv)_O_2_). GraphPad prism version 8.0.2 statistical software was used to construct box charts (median with interquartile range). The Mann–Whitney U test was used for intergroup comparison.

The ΔPCO_2_ of the n-OI group at the four time points were 9.0 (7.0–11.0), 9.0 (7.0–11.0), 9.0 (7.0–11.0), and 9.0 (7.0–11.0) mmHg (*P* = 0.84), and those of the OI group were 9.0 (8.0–12.8), 10.0 (7.0–11.0), 10.0 (8.5–12.5), and 9.0 (7.3–11.0) mmHg (*P* = 0.37). The ΔPCO_2_/C_(a−cv)_O_2_ of the n-OI group at the four time points were 2.0 (1.6–2.7), 2.1 (1.7–2.8), 2.0 (1.5–2.3), and 2.0 (1.7–2.4) mmHg/mL (*P* = 0.46), and those of the OI group were 2.1 (1.7–2.9), 2.2 (1.8–2.5), 2.1 (1.8–2.6), and 1.8 (1.6–2.5) mmHg/mL (*P* = 0.39) (Table 2), respectively. There were no statistical differences in the CO_2_-derived variables at any of the four time points (*P* > 0.05) between the n-OI and OI groups (Figure 2). Statistically significant differences were observed intragroup for pH, PO_2_, BE, Hb concentration, fraction of inspired oxygen, lactate level, fluid balance, and body temperature at all four time points (Table 2).

**Table 2:** Clinical data on ICU admission and at H4, H8, H12 post admission in n-OI and OI groups.

	**n-OI (*****n*** **= 77)**				***P-*value**	**OI (*****n*** **= 37)**				***P*** **-value**
	**ICU admission**	**H4**	**H8**	**H12**		**ICU admission**	**H4**	**H8**	**H12**	
pH	7.40 (7.35–7.45)	7.37(7.31–7.43)*	7.38(7.32–7.43)	7.39 (7.35–7.42)	< 0.01	7.39 (7.31–7.43)	7.35(7.29–7.43)	7.37(7.30–7.41)	7.39 (7.33–7.44)†	0.02
P_a_CO_2_ (mmHg)	39.0 (35.0–43.0)	39.0 (35.0–43.0)	38.0 (34.5–41.5)	37.0 (34.0–41.0)	0.15	38.0 (35.0–43.0)	38.0 (35.0–43.5)	38.0 (33.5–41.0)	35.0 (32.5–40.0)	0.10
P_a_O_2_ (mmHg)	155 (113–186)	169 (150–190)	162 (145–181)	159 (141–180)	0.04	138 (102–163)	151 (127–175)	155 (123–179)	146 (115–170)	0.24
BE (mmol/L)	−0.3 (−2.2,−1.7)	−2.2 (−5.1,−0.7)*	−2.7 (−5.3,−0.8)**	−2.2 (−3.9,−0.7)¶	< 0.01	−1.0 (−4.2–0.5)	−2.6 (−6.8–0.8)*	−3.0 (−6.5,−1.6)	−2.2 (−5.3,−0.7)	0.01
Hemoglobin (g/L)	109.0 (101.0–124.0)	116.5 (105.0–128.8)*	112.0 (102.5–129.5)	112.0 (99.3–122.5)†	0.01	116.0 (103.0–125.0)	121.0(101.0–129.0)	115.0 (99.0–127.0)	107.0 (95.5–127.5)	0.17
FiO_2_ (%)	50 (40–54)	43 (40–50)*	40 (40–50)**	40 (40–45)†¶	< 0.01	50 (40–50)	40 (40–50)	40 (40–50)	40 (40–45)¶	< 0.01
S_a_O_2_ (%)	99 (99–100)	99 (99–100)	99 (99–100)	99 (99–100)	0.15	99 (98–100)	99 (98–99)	99 (98–100)	99 (99–100)	0.18
Lactate (mmol/L)	2.6 (1.7–3.7)	3.1 (2.0–5.1)*	3.5 (2.3–5.5)**	3.3 (2.5–5.3)¶	< 0.01	3.0 (1.9–5.1)	4.1 (2.0–6.1)	4.2 (2.6–7.0)**	3.9 (2.8–6.3)	< 0.01
S_cv_O_2_ (%)	71 (61–79)	72 (62–78)	69 (64–76)	71 (59–78)	0.80	68 (61–79)	70 (63–79)	68 (61–75)	68 (62–73)	0.57
ΔPCO_2_ (mmHg)	9.0 (7.0–11.0)	9.0 (7.0–11.0)	9.0 (7.0–11.0)	9.0 (7.0–11.0)	0.84	9.0 (8.0–12.8)	10.0 (7.0–11.0)	10.0 (8.5–12.5)	9.0 (7.3–11.0)	0.37
S_a_O_2_–S_cv_O_2_ (%)	29 (20–38)	27 (21–35)	31 (23–36)	29 (21–39)	0.71	31 (22–40)	27 (20–38)	31 (21–39)	31 (26–38)	0.52
C_a-cv_O_2_ (mL/ L)	4.1 (3.1–5.4)	4.2 (3.0–5.5)	4.5 (3.7–5.4)	4.2 (3.2–5.8)	0.52	4.4 (3.8–5.5)	4.3 (3.3–5.5)	4.5 (3.4–5.9)	4.8 (3.8–5.3)	0.52
ΔPCO_2_/C_(a−cv)_O_2_ (mmHg/mL)	2.0 (1.6–2.7)	2.1 (1.7–2.8)	2.0 (1.5–2.3)	2.0 (1.7–2.4)	0.46	2.1 (1.7–2.9)	2.2 (1.8–2.5)	2.1 (1.8–2.6)	1.8 (1.6–2.5)	0.39
O_2_ER (%)	29 (20–38)	27 (21–35)	31 (23–36)	29 (21–39)	0.70	31 (23–40)	27 (20–38)	31 (22–39)	40 (27–38)	0.53
**Ventilator setting**										
Tidal volume (mL)	453 (406–498)	448 (406–496)	450 (405–500)	459 (407–500)	0.54	454 (442–500)	462 (432–497)	461 (425–501)	466 (439–507)	0.56
Frequency (/min)	15.0 (15.0–16.0)	15.0 (15.0–16.0)	15.0 (15.0–16.0)	15.0 (15.0–16.0)	0.96	15.0 (15.0–16.0)	15.0 (15.0–16.0)	15.0 (15.0–16.0)	15.0 (15.0–16.0)	0.72
PEEP (cmH_2_O)	5.0 (4.0–5.0)	5.0 (3.0–5.0)	5.0 (4.0–5.0)	5.0 (4.0–5.0)	0.84	5.0 (4.0–5.0)	5.0 (5.0–5.0)	5.0 (4.0–5.0)	5.0 (4.0–5.0)	0.78
**Fluid challenge**										
Intake (mL)	1845 (1500–2273)	640 (470–852)*	1130 (855–1483)‡**	1470 (1083–1883)¶ξ	< 0.01	1651 (1275–2370)	530 (385–749)*	1045 (885–1248)‡**	1455 (1138–1828)ξ	< 0.01
Output (mL)	1300 (1000–2000)	690 (530–893)*	1015 (818–1300)‡**	1285 (1040–1620)†ξ	< 0.01	1500 (1075–2275)	635 (490–968)*	955 (770–1349)‡**	1170 (990–1628)ξ	< 0.01
Fluid balance (mL)	261 (-25–757)	−66 (-295–250)*	88 (-180–521)‡	140 (-182–541)	< 0.01	350 (-230–900)	−90 (-398–112)*	15 (-298–327)‡	115 (-90–588)ξ	< 0.01
**VIS**	4.0 (2.0–6.8)	4.0 (2.0–8.0)	4.0 (1.9–9.0)	4.0 (1.0–9.0)	0.36	4.0 (2.0–6.0)	5.3 (1.3–10.8)	5.5 (2.0–11.8)	5.0 (2.0–11.8)	0.10
**Vital signs**										
SBP (mmHg)	105 (100–121)	105 (93–112)	103 (96–112)	104 (98–113)	0.06	105 (92–116)	103 (94–114)	103 (95–113)	108 (102–115)	0.24
DBP (mmHg)	60 (54–68)	57 (53–63)	58 (54–64)	59 (54–64)	0.10	59 (54–66)	59 (55–65)	55 (51–64)	58 (51–66)	0.16
MAP (mmHg)	76 (68–85)	72 (68–79)	72 (69–78)	73 (67–79)	0.09	73 (66–80)	75 (68–79)	74 (65–80)	73 (70–81)	0.76
CVP (mmHg)	8 (7–11)	8 (7–10)	8 (6–10)	7 (6–10)	0.14	8 (5–10)	8 (6–9)	8 (6–10)	8 (6–11)	0.86
Temperature (°C)	37.0 (36.0–37.0)	36.7 (36.5–37.0)*	37.0(36.5–37.3)‡**	37.1 (36.6–37.4)¶	< 0.01	37.0 (36.0–37.0)	36.5 (36.25–37.0)	36.8 (36.5–37.2)**	37.2 (37.0–37.6)¶	< 0.01
Respiratory rate (/min)	15 (15–16)	15 (15–16)	15 (15–16)	15 (15–16)	0.89	15 (15–16)	15 (15–16)	15 (15–16)	15 (15–16)	0.75
Heart rate (/min)	85 (78–91)	83 (76–92)	83 (74–92)	81 (74–89)	0.22	89 (79–99)	86 (75–99)	84 (71–96)	84 (80–96)	0.42

*P_a_CO_2_, arterial partial pressure of carbon dioxide; P_a_O_2_, arterial partial pressure of oxygen; BE, base excess; FiO_2_, fraction of inspiration O_2_; S_a_O_2_, arterial oxygen saturation; S_cv_O_2_, central venous oxygen saturation; ΔPCO_2_, central venous-to-arterial carbon dioxide difference; ΔPCO_2_/C_(a-cv)_O_2_, ΔPCO_2_ to arterial-central venous oxygen content difference rate; O_2_ER, oxygen extraction ratio; VIS, vasoactive-inotropic score; SBP, systolic pressure; DBP, diastolic pressure; MAP, mean arterial pressure; CVP, central venous pressure. Continuous variables are represented by median (IQR). Repeated-measures ANOVA was used for intragroup comparison*.

* *P < 0.05 for groups H0 vs. H4*,

** *P < 0.05 for groups H0 vs. H8*,

¶ *P < 0.05 for groups H0 vs. H12*,

‡ *P < 0.05 for groups H4 vs. H8*,

† *P < 0.05 for groups H4 vs. H12*,

ξ *P < 0.05 for groups H8 vs. H12*.

### The Prognostic Value of CO_2_-Derived Variables

Regarding specific time point variables, ΔPCO_2_, ΔPCO_2_/C_(a−cv)_O_2_, lactate and BE had no predictive value (Table 3). Comparison of the subgroup OI-2 to the n-OI group demonstrated that lactate and BE had limited predictive value (Table 4). After adjusting for age (median, ≤ 58, >58 years old) and gender, H8 BE (odds ratio = 1.37, 95% CI: 1.08–1.74, *P* = 0.009) was a risk factor for H48 organ dysfunction after CPB.

**Table 3:** Area under the ROC curve of variables grouped into n-OI and OI.

**Variable**	**AUC**	**Standard error**	***P*** **-value**	**95% CI**
ICU admissionΔPCO_2_	0.571	0.072	0.334	0.430–0.711
H4ΔPCO_2_	0.514	0.076	0.843	0.366–0.663
H8ΔPCO_2_	0.588	0.072	0.230	0.447–0.729
H12ΔPCO_2_	0.566	0.070	0.366	0.429–0.704
ICU admission ΔPCO_2_/C_(a−cv)_O_2_	0.510	0.075	0.892	0.363–0.658
H4ΔPCO_2_/C_(a−cv)_O_2_	0.479	0.074	0.776	0.333–0.625
H8ΔPCO_2_/C_(a−cv)_O_2_	0.588	0.071	0.236	0.449–0.728
H12ΔPCO_2_/C_(a−cv)_O_2_	0.443	0.076	0.448	0.295–0.592
ICU admission Lactate	0.586	0.063	0.150	0.463–0.710
H4 Lactate	0.569	0.063	0.254	0.446–0.691
H8 Lactate	0.567	0.060	0.265	0.449–0.685
H12 Lactate	0.574	0.063	0.219	0.451–0.697
ICU admission Base excess	0.608	0.058	0.069	0.494–0.722
H4 Base excess	0.578	0.059	0.190	0.463–0.693
H8 Base excess	0.594	0.058	0.112	0.481–0.708
H12 Base excess	0.544	0.061	0.462	0.423–0.664

*ΔPCO_2_, central venous-to-arterial carbon dioxide difference; ΔPCO_2_/C_(a−cv)_O_2_, ΔPCO_2_ to arterial-central venous oxygen content difference rate. SPSS 20 statistical software*.

**Table 4:** Area under the ROC curve of variables grouped into n-OI and OI-2.

**Variable**	**AUC**	**Standard error**	***P*** **-value**	**95% CI**
ICU admissionΔPCO_2_	0.538	0.105	0.713	0.333–0.744
H4ΔPCO_2_	0.549	0.118	0.636	0.318–0.780
H8ΔPCO_2_	0.591	0.118	0.380	0.360–0.822
H12ΔPCO_2_	0.456	0.119	0.674	0.224–0.689
ICU admission ΔPCO_2_/C_(a−cv)_O_2_	0.562	0.109	0.550	0.348–0.777
H4ΔPCO_2_/C_(a−cv)_O_2_	0.670	0.100	0.104	0.473–0.866
H8ΔPCO_2_/C_(a−cv)_O_2_	0.700	0.104	0.055	0.497–0.903
H12ΔPCO_2_/C_(a−cv)_O_2_	0.557	0.119	0.581	0.324–0.791
ICU admission Lactate	0.635	0.103	0.195	0.434–0.837
H4 Lactate	0.634	0.098	0.198	0.442–0.826
H8 Lactate	0.715	0.099	0.039*	0.521–0.908
H12 Lactate	0.634	0.123	0.198	0.393–0.875
ICU admission Base excess	0.662	0.103	0.093	0.459–0.864
H4 Base excess	0.723	0.104	0.021*	0.519–0.926
H8 Base excess	0.831	0.061	0.001*	0.710–0.951
H12 Base excess	0.703	0.103	0.035*	0.501–0.904

*ΔPCO_2_, central venous-to-arterial carbon dioxide difference; ΔPCO_2_/C_(a−cv)_ O_2_, ΔPCO_2_ to arterial-central venous oxygen content difference rate. SPSS 20 statistical software*,

* *P < 0.05*.

## Discussion

### Clinical Implications

We found that the ΔPCO_2_/C_(a−cv)_O_2_ and ΔPCO_2_ did not differ significantly between the two groups at different time points during the first 12 h after ICU admission. Elevated ΔPCO_2_ and ΔPCO_2_/C_(a−cv)_O_2_ were common phenomena after CPB. The ΔPCO_2_/C_(a−cv)_O_2_ and ΔPCO_2_ cannot be used to predict H48 organ dysfunction. The predictive performance of lactate and BE was significantly enhanced as the number of dysfunctioning organs increased, although it was limited.

To the best of our knowledge, this study is the first to analyze the association of both ΔPCO_2_ and ΔPCO_2_/C_a−cv_O_2_ on 48 h-organ dysfunction after adult cardiac surgery. In our study, the ΔPCO_2_ value, CPB time, aortic clamping time, and sequential organ failure score at ICU admission were similar to Guinot et al. study (16). They also found that ΔPCO_2_ is not predictive of postoperative complications or mortality. Their research did not provide information on the tidal volume and pCO_2_, which are thought to affect the accuracy of ΔPCO_2_ (17). Our study has very specific ventilator settings, which make up for this deficiency. Additionally, we found that rewarming, Hb dilution, and pH change were universal after CPB, which might have affected the accuracy of our estimations (18, 19). According to the literature, pCO_2_ may be elevated due to the accumulation of CO_2_ in tissues caused by reperfusion after CPB (20). Another study with negative results was reported by Morel et al. (21), who believe that ΔPCO_2_ is difficult to interpret due to the sudden variation of many parameters interfering with tissue perfusion. Such studies with negative results may have a common feature that outcomes may occur before measurement such as vasoplegia, heart failure, and acute renal failure. Furthermore, the complication itself may lead to an increased ΔPCO_2_ and, according to the time of measurement, the arrow of causation could be reversed (22). In our study, the median sequential organ failure score was 5, 4, and 3 at ICU admission, and 24 and 48 h post admission, respectively. Therefore, some of the organ dysfunction was suspected to have occurred during surgery. However, we believe that outcomes to be reliable as organ dysfunction generally results in poor outcomes (prolonged ICU stay, prolonged hospital stay).

A review of the literature also found studies with positive results. Mukai et al. (23) found that the ΔPCO_2_ at the end of cardiac surgery was a moderate predictor of postoperative complications (area under the curve [AUC]: 0.731; 95% confidence interval [CI] 0.588–0.874), at a 6.8 mmHg cut off with a sensitivity of 72.0% and specificity of 70.6%. Complications are defined as MMOM (major organ morbidity and mortality) (24), as follows: death, stroke requiring drug treatment, renal failure requiring dialysis, prolonged mechanical ventilation (more than 48 h postoperatively), re-operation, and deep sternal infection. It is worth noting that measurements were obtained at the end of surgery and the definition of complications were easy to identify. Hence, we cannot exclude that perioperative ΔPCO_2_ could be predictive of outcome (21). A previous study reported that ΔPCO_2_/C_(a−cv)_O_2_ is not associated with respiratory quotient (25). In our study, ΔPCO_2_/C_(a−cv)_O_2_ was not associated with the existence of tissue hypoxia effectively because of its poor relationship with lactate (R^2^=0.067). ΔPCO_2_/C_(a−cv)_O_2_ was grouped according to whether it was ≥1.4 mmHg/mL, and no statistical significance were detected in the length of the ventilator, LOS in ICU, or the 24 h and 48 h sequential organ failure score (data not provided). That is probably largely because of the influence of CPB on the activation of inflammation *in vivo*, ischemia-reperfusion injury, myocardial suppression, and other factors, thus, a microcirculation disorder exists that can last for 72 h (26). From our research, we have observed many factors that affect CO_2_-derived indicators after cardiac surgery in the real world, including CBP itself (27), translocation, endothelial dysfunction (28, 29), and microcirculation alterations (30) may directly or indirectly cause complications, making the interpretation of the results more complicated.

Hyperlactatemia is common after cardiopulmonary bypass, and it can be influenced by many factors. Patients undergoing CPB are exposed to the “elution effect,” i.e., the release of lactic acid from the lungs (31) and the liver due to organ malfunction associated with CPB (32). A recent study has demonstrated that hyperlactatemia may be also in relation to tissue metabolic uncoupling (33). Therefore, lactic acid does not always reflect tissue hypoxia. The predictive value of lactate after CPB surgery is limited. This study demonstrated that postoperative lactic acidemia is common, with progressively decreasing levels of lactic acid observed in both groups. After adjustment for confounding factors, compared to ΔPCO_2_ and ΔPCO_2_/C(a-cv)O_2_, lactic acid has a moderate ability to predict two or more organ dysfunction. In addition, BE is the amount of base in mmol required to titrate 1 L of whole arterial blood to a pH of 7.40, with the sample fully saturated with oxygen at 37°C and a P_a_CO_2_ of 40 mmHg. We also found that BE was a more robust predictor of H48 organ dysfunction than lactic acid, and H8 BE was a risk factor for dysfunction of two or more organs (odds ratio = 1.37), whereas lactic acid was not. A high negative value of BE may be associated with renal dysfunction, metabolic acidosis, and shock, but the factors associated with the reduced systemic BE in patients after cardiac surgery are yet to be clarified (34). We speculated that lactic acidosis might be one of the main reasons leading to the high negative value of BE because a good correlation was established between BE and lactic acid (*R2* = 0.599, *P* < 0.05), which could also explain the low susceptibility of BE to interference from other factors.

### Methodological Considerations

This study has some limitations. First, this was a single-center study, and its results may not be generalizable. Second, the sample size was relatively small, and no deaths occurred among the study patients. Third, because the group classification was based solely on the occurrence of organ dysfunction, a misclassification bias may have occurred. However, our subgroup analysis was based on the number of failing organs and resulted in similar conclusions. Fourth, P_cv−a_CO_2_/C_(a−cv)_O_2_ cannot replace P_mv−a_CO_2_/C_(a−cv)_O_2_ (mv: mixed venous blood) and might have underestimated the CO_2_ exchange from splanchnic circulation. Finally, it was not easy to detect when the outcome of interest, i.e., organ dysfunction, first occurred relative to the measurement. Continuous measurements or change over time may have more prognostic value than a single time point. However, the dynamic changes of ΔPCO_2_ at t0 and T6 time points were not found to be associated with postoperative complications or mortality in children after CPB (16).

## Conclusion

In conclusion, ΔPCO_2_ and ΔPCO_2_/C_(a−cv)_O_2_ cannot be used as reliable indicators to predict the occurrence of organ dysfunction at 48 h after CBP, which is due to the complex pathophysiological processes after CBP.

## Data Availability Statement

The original contributions presented in the study are included in the article/supplementary material, further inquiries can be directed to the corresponding author/s.

## Ethics Statement

The studies involving human participants were reviewed and approved by Ethics Committee of Taizhou Hospital, Zhejiang Province. The patients/participants provided their written informed consent to participate in this study. Written informed consent was obtained from the individual(s) for the publication of any potentially identifiable images or data included in this article.

## Author Contributions

SZ: data curation, investigation, resources, writing—original draft, validation, and funding acquisition. DZ: data curation, investigation, and methodology. X-QC: data curation, investigation, methodology, and software. Y-PJ, L-ZQ, W-YY, and W-YZ: data curation, investigation, and resources. C-GW: data curation and formal analysis. Q-MZ: data curation and resources. T-HT: design of the study and performed data synthesis of the revised stage. R-HL: conceptualization, data curation, formal analysis, funding acquisition, software, validation, writing—review, and editing. All authors have read and approved the manuscript.

## Funding

This study was supported by the Medical and Health Science and Technology Project, Zhejiang Province (2017KY163). The funders had no role in study design, data collection and analysis, decision to publish, or preparation of the manuscript.

## Conflict of Interest

The authors declare that the research was conducted in the absence of any commercial or financial relationships that could be construed as a potential conflict of interest.

## Publisher's Note

All claims expressed in this article are solely those of the authors and do not necessarily represent those of their affiliated organizations, or those of the publisher, the editors and the reviewers. Any product that may be evaluated in this article, or claim that may be made by its manufacturer, is not guaranteed or endorsed by the publisher.

## Abbreviations

ΔPCO_2_/C_(a−cv)_O_2_: Arterial-venous O_2_ content difference ratio
CO_2_: carbon dioxide
CPB: cardiopulmonary bypass
Hb: hemoglobin
ICU: intensive care unit
n-OI: non-organ dysfunction group
OI: organ dysfunction group
ΔPCO_2_: venous-to-arterial CO_2_ difference.
